# Age Related Prevalence of Mild Cognitive Impairment in Type 2 Diabetes Mellitus Patients in the Indian Population and Association of Serum Lipids With Cognitive Dysfunction

**DOI:** 10.3389/fendo.2021.798652

**Published:** 2021-12-31

**Authors:** Arpita Chakraborty, Sumukha Hegde, Samir K. Praharaj, Krishnananda Prabhu, Chhaya Patole, Ashok K. Shetty, Shreemathi S. Mayya, Raviraj V. Acharya, H. Manjunath Hande, M. Mukhyaprana Prabhu, Dinesh Upadhya

**Affiliations:** ^1^ Centre for Molecular Neurosciences, Kasturba Medical College Manipal, Manipal Academy of Higher Education, Manipal, India; ^2^ Department of General Medicine, Kasturba Medical College Manipal, Manipal Academy of Higher Education, Manipal, India; ^3^ Department of Psychiatry, Kasturba Medical College Manipal, Manipal Academy of Higher Education, Manipal, India; ^4^ Department of Biochemistry, Kasturba Medical College Manipal, Manipal Academy of Higher Education, Manipal, India; ^5^ Mass Spectrometry Facility, Institute For Stem Cell Science and Regenerative Medicine, Centre for Cellular and Molecular Platforms Campus, National Centre for Biological Sciences, Bangalore, India; ^6^ Institute for Regenerative Medicine, Texas Agricultural and Mechanical (A&M) Health Science Center College of Medicine, College Station, TX, United States; ^7^ Department of Molecular and Cellular Medicine, Texas Agricultural and Mechanical (A&M) Health Science Center College of Medicine, College Station, TX, United States; ^8^ Department of Data Science, Prasanna School of Public Health, Manipal Academy of Higher Education, Manipal, India

**Keywords:** cognitive dysfunction, type 2 diabetes mellitus, Montreal Cognitive Assessment test, lipids, mild cognitive impairment, untargeted and targeted lipidomics

## Abstract

The magnitude of type 2 diabetes mellitus (T2DM) is ever-increasing in India, and at present, ~77 million people live with diabetes. Studies have established that T2DM increases the risk of neurodegenerative disorders. This study aimed to determine the age-related prevalence of mild cognitive impairment (MCI) in T2DM patients in the Indian population and to identify link between cognitive dysfunction in T2DM patients and serum lipid composition through untargeted and targeted lipidomic studies. Using a cross-sectional study, we evaluated 1278 T2DM patients with Montreal cognitive assessment test (MoCA) and digit symbol substitution test (DSST) for cognitive functions. As per MoCA, the prevalences of MCI in T2DM patients in age groups below 40, 41-50, 51-60, 61-70, 71-80 and 81-90 years were 13.7, 20.5, 33.5, 43.7, 57.1 and 75% with DSST scores of 45.8, 41.7, 34.4, 30.5, 24.2 and 18.8% respectively. Binomial logistic regression analysis revealed serum HbA1c ≥ 7.51, duration of T2DM over 20 years, age above 41 years, and females were independent contributors for cognitive dysfunction in T2DM patients. Preliminary studies with untargeted lipidomics of the serum from 20 T2DM patients, including MCI and normal cognition (NC) group, identified a total of 646 lipids. Among the identified lipids, 33 lipids were significantly different between MCI and NC group, which comprised of triglycerides (TGs, 14), sphingolipids (SL, 11), and phosphatidylcholines (PC, 5). Importantly, 10 TGs and 3 PCs containing long-chain polyunsaturated fatty acids (PUFA) were lower, while 8 sphingolipids were increased in the MCI group. Since brain-derived sphingolipids are known to get enriched in the serum, we further quantified sphingolipids from the same 20 serum samples through targeted lipidomic analysis, which identified a total of 173 lipids. Quantitation revealed elevation of 3 species of ceramides, namely Cer (d18:1_24:1), Hex1Cer (d16:0_22:6), and Hex2Cer (d28:1) in the MCI group compared to the NC group of T2DM patients. Overall, this study demonstrated an age-related prevalence of MCI in T2DM patients and highlighted reduced levels of several species of PUFA containing TGs and PCs and increased levels of specific ceramides in T2DM patients exhibiting MCI. Large-scale lipidomic studies in future could help understand the cognitive dysfunction domain in T2DM patients, while studies with preclinical models are required to understand the functional significance of the identified lipids.

## Introduction

The magnitude of type 2 diabetes mellitus (T2DM) is increasing globally, with a rapid increase in prevalence is seen in low and middle-income countries (1). Currently, ~77 million people live with diabetes in India, which is expected to reach 134 million by 2045 (2). Uncontrolled serum glucose levels for extended durations are associated with retinopathy, nephropathy, and cardiovascular, cerebrovascular, and peripheral vascular diseases, leading to high morbidity and mortality rates in T2DM. Apart from these major morbidities, recently, mild cognitive impairment (MCI) in T2DM is gaining much attention as MCI can enhance the risk for developing dementia (3–5). The primary cognitive domains associated with T2DM are working memory, verbal fluency, immediate and delayed recall, visual perception, psychomotor speed, executive control, auditory, memory and processing speed, attention, etc. (6). A recent meta-analysis data from 12 studies revealed the global prevalence of MCI at 45% (7) with the lowest reported prevalence of 21.8% (8) and the highest prevalence of 67.5% (9) among 256 and 400 T2DM patients evaluated, respectively (7). The majority of the previous studies have performed cognitive analyses in T2DM patients either above 60 or 65 years. There have been no detailed large-scale studies on the prevalence of MCI in T2DM patients at different ages.

Lipids constitute half of the brain’s dry weight and play critical roles as structural and functional components of the brain (10–12). The extent of changes in lipid composition of the developmental and dysfunctional brain is still evolving (12). Such data could give critical inputs for interventional strategies through targeted dietary supplementation or restricted diet to prevent the onset of MCI or to slower the development of MCI into dementia. Sphingolipids are a class of lipids abundant in the brain compared to its concentration in plasma as they constitute ~22% dry weight of the human brain white matter while it accounts for 5% dry weight of plasma (13–15). Certain sphingolipid species levels are 20-90 folds higher in the brain than plasma (16). Thus evaluation of sphingolipids in the serum of T2DM patients with MCI and NC through targeted lipidomics could provide inputs on changes in some key lipid components, which could be essential for preserving neural functions.

The present cross-sectional study evaluated the prevalence of MCI in 1278 T2DM patients, including all ages in the south Indian population, using Montreal cognitive assessment test (MoCA) and digit symbol substitution test (DSST). Data were stratified on the different analytical basis and correlated with their socio-demographic and clinical features. Binomial regression analysis was performed to identify the factors influencing MCI in T2DM patients. Further, in a preliminary evaluation, the study compared the serum lipid composition of 20 T2DM patients through untargeted lipidomics, which included age-matched MCI and NC groups. Further, sphingolipid species are quantified in the same 20 T2DM patients using targeted lipidomics to identify significantly altered sphingolipids in T2DM patients with MCI.

## Methods

### Study Population

This cross-sectional study was conducted at a tertiary care hospital in coastal Karnataka, South India, following institutional ethics committee approval and voluntary consent from the participants (IEC 715/2018). Confirmation of T2DM in each patient was based on the evaluation of fasting and postprandial blood sugar and disease history. For the prevalence of MCI, 1278 T2DM patients of all ages were included. Severely sick T2DM patients and those with visual impairment, confusion, delirium, and less than 5 years of formal education were excluded from the study. For the untargeted and the targeted lipidomics studies, 20 patients (10 with MCI and 10 NC) having T2DM for ≥10 years with an age of 60 ± 5 years were considered. For the lipidomics, T2DM patients suffering from hypo and hyperthyroidism, hypertension, HIV, Vitamin B12 deficiency, kidney, liver, and other infectious diseases were excluded to avoid specific condition related changes.

### Sociodemographic and Clinical Data Collection

Sociodemographic and clinical details were obtained using a proforma for the study which comprised details about sex, age, body mass index, duration of T2DM, years of education, smoking habits, dietary habits, alcohol consumption, disease history, diabetes, medications including statin and insulin use.

### Montreal Cognitive Assessment Test

Montreal Cognitive Assessment Test (MoCA) has been used as a rapid screening tool to assess cognitive function. MoCA has been recognized as a better tool than the other cognitive tools to detect cognitive dysfunction in T2DM patients (17). Cognitive fields such as emphasis and attention, executive functions, memory and vocabulary, visuo-constructional capacity, conceptual reasoning, calculations, and orientations were assessed using this test. MOCA detects 90% of subjects with cognitive dysfunction (18).

The MoCA test is a one-page 30-point test performed in approximately 10 minutes for a patient. The test is available in multiple languages. This test assesses different cognitive domains such as short-term memory recall task, visuospatial abilities, different aspects of executive functions, a phonemic fluency task, and a two-item verbal abstraction task, attention, concentration, and working memory. These functions were evaluated using a sustained attention task, a serial subtraction task, and digits forward and backward. Language assessment was performed using a three-item confrontation naming task with low-familiarity animals, repetition of two syntactically complex sentences, and fluency as mentioned above. Orientation to time and place was evaluated by asking the subject for the date and the city where the test occurred. Each of these tests carried specific scores, and a total possible score of 30 points was considered normal. The cut-off value used in MOCA to detect MCI was < 26. For a person who has 12 years or less of formal schooling, one extra point was added. MoCA was used in two languages, i.e., English and Kannada, for the required patients.

### Digit Symbol Substitution Test

Digit symbol substitution test (DSST) is a neuropsychological test that assesses mental speed. DSST is a reliable tool and is affected little by language and education (19). This test is a simple paper-and-pencil cognitive test, consists of digit-symbol pairs. Under each digit, the subject was asked to write down the corresponding symbol as fast as possible. The number of correct symbols within the allowed time of 120 seconds was measured, and the number of correct matches in the allotted time provided a score of cognition. Higher scores implied better performance by the individuals. Following DSST, the Digit Symbol-Coding test was paired, and free recall of the symbols was assessed.

### Blood Collection and Evaluation of Biochemical Variables

Once patients were identified as T2DM and cognitive evaluations were done, we collected 5ml of blood samples in vacutainer tubes. The serum was separated by centrifugation and aliquoted and stored at -80°C until analysis. Clinical variables such as fasting blood sugar (FBS), postprandial blood sugar (PPBS), and glycated hemoglobin (HbA1c) levels along with fasting lipid profile parameters such as total cholesterol (TC), triglycerides (TG), HDL cholesterol, LDL cholesterol, TC/HDL and Non-HDL cholesterol levels were also evaluated using standard laboratory methods. Both Vitamin B12 and TSH levels were estimated by electro-chemiluminescence immunoassay (ECLIA).

### Untargeted Lipidomics and Targeted Sphingolipidomics

A ten µl serum sample in a 1.5 ml capacity low retention Eppendorf tube was spiked with 10 µl of the splash standard. An equal volume of methanol, chloroform, and 0.89% potassium chloride was added, vortexed for a minute, incubated at 37°C for 10 minutes, and centrifuged at 10000 rpm at 4°C for 5 minutes. The lower organic phase was separated into a 1-5ml clean test tube. Equal volumes of chloroform and 0.89% potassium chloride were added to the original Eppendorf tube containing the upper layer. This step was repeated, and a lower organic phase was collected into the same 1.5 mL test tube. Solvents were evaporated to dryness in gentle nitrogen stream or under vacuum and the residue was reconstituted in 200 uL of MeOH. Five uL was injected into the Acclaim ™ C30 column in a Thermo Q-Exactive Coupled to a Dionex UPLC chromatographic system of LCMS model at a flow rate of 300µl/min with the gradient mobile phase containing ammonium formate with formic acid in acetonitrile/water and ammonium formate with formic acid in isopropanol/acetonitrile. Lipids were detected using Orbitrap mass spectrometer in positive and negative ion polarity mode operated in MS mode scan range (m/z) 200-1200 with maximum injection time (MS) of 50 ms. The detector type in MS/MS is higher-​energy collisional dissociation assisted with high collision energy ion fragmentation with fragmentation energy of 25-40 and MS/MS scan range (m/z) 50-1200.

### Data Analysis Using Lipid Search v. 4.2

LipidSearch software v 4.2 (Thermo, CA) was used to identify and quantify lipids from 18 major lipid classes in the database, based on accurate precursor mass and characteristic fragments. Lipid identification was based on an MS/MS match. The mass tolerances for the precursor and fragment were 5 ppm and 5ppm, respectively. A retention time shift of 0.15 min was allowed for quantitation and alignment. The criteria to eliminate the false positives were lipid species-dependent. In addition, since ammonium acetate was used in the mobile phase, the adducts of + H, + NH4 were used in the positive mode search, and—H, + CH3COO in the negative mode.

### Statistical Analysis

Data were analyzed using GraphPad Prism software. Prevalence data from MoCA were expressed in percentage. Scores of DSST were expressed in mean ± SEM as not much difference was observed between the mean and median. Median values were also considered for DSST scores in each age group. Percentage and median with Q1 and Q3 were used for comparison of socio-demographic and clinical characteristics of T2DM patients with NC and MCI. Mann Whitney test was used to find the association between the variables in T2DM patients with NC and MCI. Chi square test was applied to find the association between the categorical parameters in T2DM patients with NC and MCI. Binomial backward logistic regression analysis was done to examine the factors related to the MCI. For untargeted lipidomics, all values were log-2-transformed to achieve normalization. Fold changes were calculated for all the lipids. For targeted lipidomics, concentrations were calculated based on standards and expressed as µg/ml. Results between T2DM MCI patients and NC patients were compared using unpaired t - tests. Significance was considered when p values were <0.05.

## Results

### Prevalence of MCI in T2DM Patients at Different Ages

The clinical and demographic details of all the patients recruited in this study are summarized in **Table 1**. A total of 1278 T2DM patients were enrolled in the study with a median age group of 58 years. Among them, 821 patients were males, and 457 patients were females. Based on MoCA scores, MCI was found in 458 (35.8%) T2DM patients. Among males, 28.1% had MCI, while 49.6% among females had MCI. Age-wise stratification of MCI data with respect to DSST score was presented in **Table 1**. Furthermore, as there was no gold standard to define MCI based on DSST scores, we compared MoCA scores with DSST scores in the same population. Evaluation of DSST analysis indicated that among 1278 patients, the DSST score (Mean ± SEM) was 34.0 ± 0.5 (median 33). Among 821 male T2DM patients, the DSST score was 36.49 ± 0.63 (median 37), and among 457 female T2DM patients, the DSST score was 29.57 ± 0.81 (median 25). The evaluation of the linear correlation between MoCA scores and DSST scores in the T2DM population demonstrated a strong positive correlation with a spearman r-value of 0.73, with a 95% confidence interval of 0.7 to 0.75 and with a two-tailed p-value <0.0001.

**Table 1:** Age wise stratified data of MCI and respective DSST scores in T2DM patients.

Age (years)	Total patients	MCI^†^ (%)	DSST score, Mean ± SEM (Median)		
			Total	T2DM patients with NC	T2DM patients with MCI
All age groups	1278	35.8	34.0 ± 0.5 (33)	42.4 ± 0.6 (41)	19.0 ± 0.4 (17)**
				n = 820	n = 458
< 40	73	13.7	45.8 ± 2.4 (44)	49.5 ± 2.4 (47)	22.2 ± 3.5 (18.5)**
				n = 63	n = 10
41-50	250	20.5	41.7 ± 1.2 (40)	46.6 ± 1.2 (43)	21.9 ± 1.5 (20)**
				n = 199	n = 51
51-60	411	33.5	34.4 ± 0.8 (35)	42.1 ± 0.9 (40)	19.2 ± 0.7 (18)**
				n = 273	n = 138
61-70	388	43.7	30.5 ± 0.8 (28)	39.2 ± 1.0 (40)	19.3 ± 0.7 (17)**
				n = 219	n = 169
71-80	140	57.1	24.2 ± 1.2 (19.5)	33.6 ± 1.9 (33)	17.2 ± 0.9 (15)**
				n = 60	n = 80
81-90	16	75	18.8 ± 2.9 (13.5)	36.5 ± 3.5 (36)	12.8 ± 1.2 (11.5)*
				n = 4	n = 12

^†^Based on MoCA scores.

**p < 0.0001, *p < 0.01, all comparisons were made between T2DM patients with MCI versus respective NC group.

### Binomial Logistic Regression Analysis Showing the Potential Association of MCI With Other Clinical and Socio-Demographic Factors

The clinical and demographic details of all the patients recruited in this study were summarized in (**Table 2**). Based on MoCA scores, MCI was found in 458 (35.8%) of T2DM patients. Mann Whitney test analysis showed that the median age (p<0.0001), duration of T2DM (p=0.011) and glycated hemoglobin (p=0.014) were higher, and years of formal education (p<0.0001) was lower in patients with MCI. Chi square test showed that a higher proportion of females had MCI, whereas alcohol users (p=0.034) and vegetarians (p<0.0001) were proportionally less in those with MCI. Mean TG levels (p=0.045) and TC/HDL ratio (p=0.017) were higher among MCI patients, whereas mean HDL cholesterol levels (p=0.015) were lower. No difference was found between the groups on TC and LDL cholesterol levels. The median DSST scores were significantly lower among those with MCI (p<0.0001).

**Table 2:** Comparison of socio-demographic and clinical characteristics of T2DM patients with NC and MCI (n=1278).

Name of variables	All T2DM patients (n = 1278)	T2DM patients with NC (n = 820)	T2DM patients with MCI (n = 458)	p value*
Age (years), Md (Q_1_,Q_3_)	58 (50,66)	56 (48,63)	62 (56,69)	**<0.0001**
Gender, n (%)				
Male	821 (64.2)	590 (72)	231 (28)	**<0.0001**
Female	457 (35.8)	230 (50.4)	227 (49.6)	
Smoking use, n (%)				
Yes	163 (14.6)	113 (69.3)	50 (30.7)	0.162
No	1115 (85.4)	707 (63.4)	408 (36.6)	
Alcohol use, n (%)				
Yes	370 (29)	254 (68.6)	116 (31.4)	**0.034**
No	908 (71)	566 (62.3)	342 (37.7)	
Statins, n (%)				
Statin users	506 (43.8)	318 (76.7)	188 (23.3)	.438
Statin non-users	772 (56.2)	502 (65.02)	270 (34.98)	
Insulin, n (%)				
Insulin users	275 (21.5)	168 (61)	107 (39)	
Insulin non-users	1003 (78.5)	652 (51)	351 (49)	.256
Diet, n (%)				
Vegetarian	392 (30.67)	281 (71.7)	111 (28.3)	**<0.0001**
Non-vegetarian	886 (69.33)	539 (60.8)	347 (39.2)	
BMI (Kg/m^2^), Md (Q_1_,Q_3_)	25.2 (22.9, 27.91)	25.2 (23, 27.9)	25 (22.28, 27.96)	0.25
Education years, Md (Q_1_,Q_3_)	10 (7,12)	12 (10, 15)	5 (5, 8)	**<0.0001**
Duration of T2DM (years), Md (Q_1_,Q_3_)	7 (3,13)	7 (3, 12)	8 (4, 15)	**0.011**
HbA_1_c (mg/dl), Md (Q_1_,Q_3_)	7.6 (6.77,9.02)	7.5 (6.7, 9)	7.8 (6.87, 9.22)	**0.014**
FBS (mg/dl), Md (Q_1_,Q_3_)	139 (117,179)	139 (117, 178)	142 (117, 183)	0.51
PPBS (mg/dl), Md (Q_1_,Q_3_)	204 (162,270)	202.5 (160, 267)	207 (168, 286)	0.09
TC (mg/dl), Md (Q_1_,Q_3_)	163.5 (135,192)	163.5 (134,191.75)	163.5 (136, 193)	0.80
TG (mg/dl), Md (Q_1_,Q_3_)	143 (106,188)	138 (102, 192.75)	148 (119, 183.25)	**0.045**
HDL (mg/dl), Md (Q_1_,Q_3_)	39 (33,46)	39 (33, 47)	38 (33, 42)	**0.015**
LDL (mg/dl), Md (Q_1_,Q_3_)	91 (67,117.6)	91 (66.62, 116)	92 (67, 119)	0.61
TC/HDL, Md (Q_1_,Q_3_)	4.16 (3.36,5.15)	4.09 (3.28, 5.11)	4.29 (3.45, 5.27)	**0.017**
Non HDL (mg/dl), Md (Q_1_,Q_3_)	123 (95, 150.25)	123 (97, 152)	121.5 (92.75, 149.25)	0.35
DSST, Md (Q1, Q3)	33 (19,45)	41 (31, 51)	17 (13, 23)	**<.0001**

*Mann-Whitney test/Chi square test.

HbA1c, glycated hemoglobin; FBS, fasting blood sugar; PPBS, post prandial blood sugar; TC,total cholesterol; TG, triglyceride; HDL, High density lipoprotein cholesterol; LDL, Low Density Lipoprotein cholesterol.

Values in bold indicate significant difference between T2DM patients with NC and MCI.

For understanding the potential association of gender, age, diet, glycated hemoglobin, insulin use, alcohol consumption, statin use, duration of diabetes and formal education with MCI in T2DM patients, we have performed binomial backward logistic regression analysis (**Table 3**). This demonstrated greater risk of MCI among females (OR 1.52, 95% CI 1.11 to 2.08, p=0.009), those with higher age (>60 years OR 9.92, 95% CI 4.28 to 23, p<0.0001; 41-60 years OR 3.58, 95% CI 1.58 to 8.13, p=0.002), raised glycated hemoglobin (≥ 7.51 mg/dl OR 1.72, 95% CI 1.12 to 2.64, p=0.013), longer duration of T2DM (>20 years OR 2.04, 95% CI 1.09 to 3.81, p=0.025), and lower education (5-9 years OR 46.12, 95% CI 25.47 to 83.51, p <0.0001; 10-14 years OR 4.21, 95% CI 2.29 to 7.72, p<0.0001). These variables together explained 49.8% variance in MCI in T2DM patients (Nagelkerke R^2^ =.498) (p<.0001). However, none of the lipoprotein parameters correlated with MCI.

**Table 3:** Binomial logistic regression analysis showing the factors related to mild cognitive impairment in T2DM patients (N= 1278).

Name of variables	Unstandardized coefficients		Exp B	95% CI for Exp B		P value
	B	SE		Lower	Upper	
**Gender**						
Male	Reference					
Female	0.42	0.16	1.52	1.11	2.08	**0.009**
**Age**						
≤ 40 years	Reference					
41-60 years	1.27	0.42	3.58	1.58	8.13	**0.002**
> 60 years	2.29	0.43	9.92	4.28	23	**<0.0001**
**HbA_1c_ (mg/dl)**						
≤ 6.5	Reference					
6.51-7.5	0.13	0.22	1.14	0.72	1.80	0.57
≥ 7.51	0.54	0.23	1.72	1.12	2.64	**0.013**
**HDL (mg/dl)**						
≥ 61	Reference					
41-60	0.24	0.41	1.27	0.56	2.85	0.56
≤ 40	0.80	0.41	2.22	0.99	4.98	0.05
**Duration of T2DM**						
0-10 years	Reference					
11-20 years	-0.12	0.19	0.88	0.61	1.27	0.50
> 20 years	0.71	0.32	2.04	1.09	3.81	**0.025**
**Education**						
≥ 15 years	Reference					
10-14 years	1.43	0.31	4.21	2.29	7.72	**<0.0001**
5-9 years	3.83	0.30	46.12	25.47	83.51	**<0.0001**

Nagelkerke R^2^ = .498, χ^2^value= 576.69, p < .0001. The odds of developing MCI in female T2DM patients was 1.52 times the odds of males. The odds of developing MCI in T2DM patients of age group 41-60 years was 3.58 times whereas the odds of developing MCI in T2DM patients of age group > 60 years was 9.92 times compared to <40 age group. The odds of developing MCI in T2DM patients with HbA1c levels > 7.51 levels was 1.72 times, duration of T2DM >20 years was 2.04 times and educational qualification of 5-9 years and 10-14 years were 46.12 times and 4.21 times respectively.

### Gender Influence on the Observed Alteration in Lipid Profile in T2DM Patients Exhibiting MCI

Among 458 T2DM patients with MCI, 231 were males and 227 were females. Comparison of serum lipid profile parameters among male and female T2DM patients with MCI demonstrated significantly higher levels (p<0.0001) of total cholesterol and HDL cholesterol among females compared to males (**Table 4**). Total triglycerides, LDL cholesterol and non HDL cholesterol levels were similar between the groups. Also, DSST scores were similar between the groups.

**Table 4:** Comparison of lipid profile parameters and DSST score in T2DM females and males with MCI.

Name of variables	T2DM females with MCI (n = 227) Median (Q1,Q3)	T2DM males with MCI (n = 231) Median (Q1,Q3)	P value*
TC (mg/dl)	167 (141,205)	155 (129,181)	<.0001
TG (mg/dl)	152 (124,187)	145 (114,179)	.160
HDL (mg/dl)	39 (35,46)	37 (31,41)	<.0001
LDL (mg/dl)	93 (71,124)	90 (64,114)	.115
TC/HDL	4.23 (3.35,5.39)	4.3 (3.53,5.15)	.583
Non HDL (mg/dl)	123 (90,150)	121 (93,147)	.946
DSST score	17 (13,22)	17 (13,24)	.195

*Mann Whitney test.

We also evaluated diabesity in T2DM patients in the studied population. Among 1278 T2DM patients, 1090 were non-obese and 188 were obese. Among 458 T2DM patients with MCI, 391 were non-obese and 67 were obese. Comparison of serum lipid profile parameters among obese and non-obese T2DM patients with MCI demonstrated comparable serum levels of total cholesterol, triglycerides, HDL and non HDL cholesterol (**Table 5**). Further, DSST scores were comparable between these two groups.

**Table 5:** Comparison of lipid profile parameters and DSST score in T2DM non-obese and obese patients with MCI.

Name of variables	T2DM non-obese patients with MCI (n = 391) Median (Q1,Q3)	T2DM obese patients with MCI (n = 67) Median (Q1,Q3)	P value*
TC (mg/dl)	164 (137,193)	157 (134,199)	.816
TG (mg/dl)	148 (118,184)	149 (125,180)	.792
HDL (mg/dl)	38 (33,42)	39 (35,46)	.121
LDL (mg/dl)	92 (66,120)	88 (71,118)	.853
TC/HDL	4.3 (3.48,5.32)	4.03 (3.38,5.02)	.271
Non HDL (mg/dl)	121 (93,148)	132 (89,154)	.290
DSST score	17 (13,23)	18 (14,22)	.352

*Mann Whitney test.

### Relative Quantitation of Serum Lipids

Serum lipids were evaluated in 20 T2DM patients with MCI (n=10) and NC (n=10) using an untargeted lipidomics approach to evaluate the possible correlation of lipid metabolism with cognitive dysfunction in T2DM. As TSH and vitamin B12 have been shown to influence lipid metabolism (20, 21), we measured TSH and vitamin B12 levels in all the samples used for lipidomics. Vitamin B12 levels were 633.8 ± 158.7 and 852.1 ± 201.4 and TSH levels were 1.81 ± 0.22 and 2.54 ± 0.35, respectively in NC and MCI, T2DM patients, demonstrating no significant difference between the groups.

**Figure 1:**
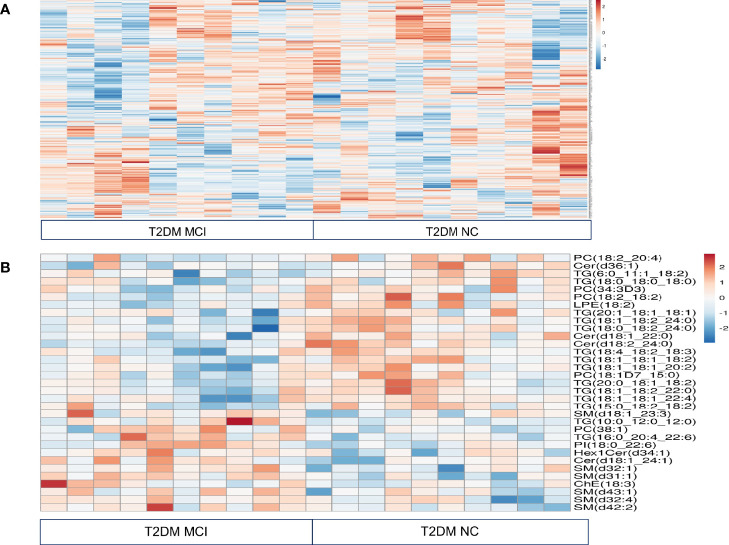
Relative quantitation of different lipids in the serum of type 2 diabetes mellitus patients with mild cognitive impairment (MCI) versus normal cognition (NC) using untargeted lipidomic analysis were presented in a heat map **(A)**. This analysis identified a total of 646 lipids in different classes. Comparison of fold change in the log 2 transformed values of T2DM NC patients with MCI patients identified significant changes in 14 species of triacylglycerols, 11 species of sphingolipids, 5 species of phosphatidylcholine, one species each of phosphatidylethanolamine, phosphatidylinositol, and cholesterol ester. These results were visualized in a heat map **(B)**.

The heat map presented the relative measurement of different lipids in the serum using an untargeted lipidomic analysis (**Figure 1A**). This analysis identified a total of 646 lipids in different classes. This included triacylglycerols (TG, 253), diacylglycerols (DG, 19), monoacylglycerols (MG, 4), phosphatidylcholine (PC, 174), phosphatidylserine (PS, 9), phosphatidylethanolamine (PE, 48), phosphatidylinositol (PI, 14), phosphatidylglycerol (PG, 4), cholesterol esters (ChE, 13), cardiolipin (CL, 4) acylcarnitine (AC, 4), and sphingolipids (100, including 35 ceramides 65 sphingomyelins). Comparison of fold change in the log 2 transformed values of T2DM NC patients with MCI patients identified significant changes in 14 species of triacylglycerols, 11 species of sphingolipids, 5 species of phosphatidylcholine, one species each of phosphatidylethanolamine, phosphatidylinositol, and cholesterol ester.

Among 14 TG species altered between T2DM with MCI and NC patients, levels of 12 species of TG specifically, TG (6:0_11:1_18:2, 18:1_18:1_18:2, p< 0.02), (18:1_18:1_20:2, 18:1_18:2_22:0 and 18:1_18:1_22:4, p< 0.03), (18:0_18:0_18:0, p<0.04), (15:0_18:2_18:2, 20:0_18:1_18:2 and 20:1_18:1_18:1, p<0.05), (18:0_18:2_24:0 and 18:4_18:2_18:3, p< 0.01) and (18:1_18:2_24:0. p<0.001), were lower in MCI patients compared to NC patients. Interestingly, out of 12 TG species exhibiting lower levels in the MCI group, 10 were polyunsaturated fatty acids. The levels of two TG species (TG 16:0_20:4_22:6, p<0.02 and 10:0_12:0_12:0, p<0.01) were higher in the MCI patients. Data were presented in the heat map (**Figure 1B**).

Among 11 sphingolipids altered, 6 species of sphingomyelins precisely SM (d18:1_23:3, p<0.05), (d32:4 and d43:1, p<0.04), (d32:1 and d42:2, p<0.02), (d31:1, p<0.001), and 2 species of ceramides, namely Cer(d18:1_24:1, p<0.003) and Hex1Cer(d34:1, p<0.05) were higher in T2DM patients with MCI compared to NC patients. However, ceramide species such as Cer, (d36:1, p<0.02), (d18:1_22:0, p<0.01) and (d18:2_24:0, p<0.001) were lower in T2DM patients with MCI compared to NC patients (**Figure 1B**).

In the PC groups, 4 PC species such as PC (18:2_20:4, 18:1D7_15:0 and 34:3D, p<0.05) as well as PC (18:2_18:2, p<0.01) were lower in T2DM patients with MCI compared to the NC group. Interestingly, out of these 4 species, 3 contained polyunsaturated fatty acids. The concentration of PC (38:1, p< 0.03) was higher in T2DM patients with MCI when compared to the NC group. Among other lipid species, the LPE (18:2, p<0.03) was significantly lower, while the PI (18:0_22:6, p<0.02) and ChE (18:3 p<0.05) were significantly higher in T2DM patients with MCI compared to NC patients (**Figure 1B**).

As triacylglycerol and sphingolipid species were significantly altered between MCI and NC groups of T2DM patients, we explored them further. As triacylglycerols are mostly derived from diet, and sphingolipids are abundant in the brain compared to their concentration in the plasma, we explored the abundance of sphingolipids in these groups.

### Quantitation of Sphingolipids Using Targeted Lipidomics

**Figure 2:**
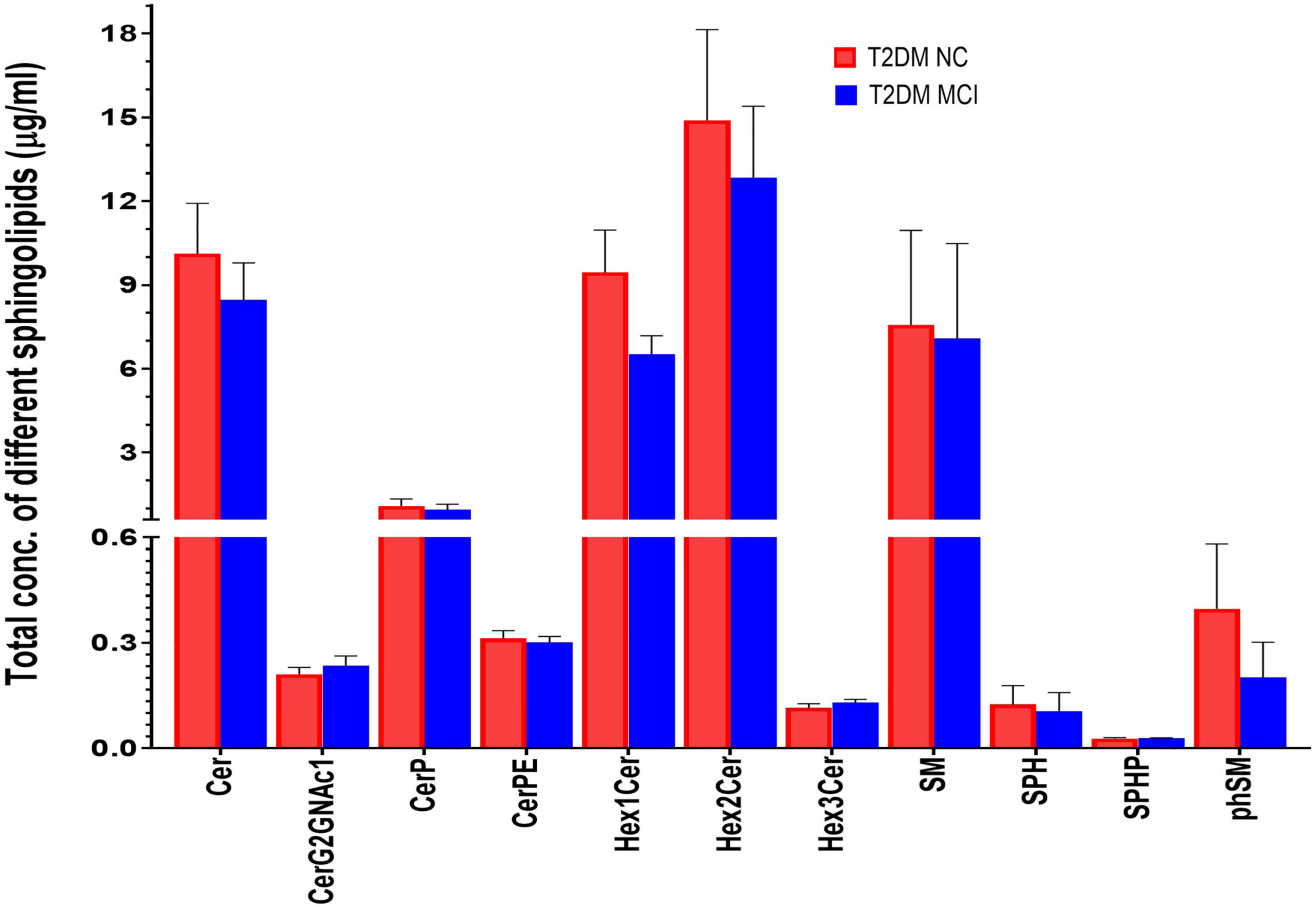
The amount of different sphingolipids in the serum type 2 diabetes mellitus patients with mild cognitive impairment (MCI) versus normal cognition (NC) were evaluated using targeted sphingolipidomic analysis and were presented in **Figure 2**. Evaluation of the total amount of different sphingolipids in MCI and NC groups revealed no significant differences between ceramides (p = 0.47), CerG2GNAc1 (p=0.46), ceramide phosphates (CerP, p=0.67), phosphorylethanolamine ceramides (CerPE, p=0.69), monohexosylceramides (Hex1Cer, p=0.09), dihexosylceramides (Hex2Cer, p=0.62), trihexosyl ceramides (Hex3Cer, p=0.35), sphingomyelins (SM, p=0.92), sphingosine (SP, p=0.8), sphingosine-1-phosphate (SPH, p=0.9) as well as phytosphingomelins (phSM, p=0.37).

The amount of different sphingolipids in the serum of T2DM patients in MCI and NC groups were evaluated using a targeted sphingolipidomic analysis. This analysis identified a total of 173 sphingolipid species which included ceramides (32), CerG2GNAc1 (6) and ceramide phosphates (6), phosphorylethanolamine ceramides (4), monohexosylceramides (22), dihexosylceramides (28), trihexosyl ceramides (2), sphingomyelins (66), sphingosine (4), sphingosine-1-phosphate (1), and phytosphingomyelins (2). The evaluation of the total amount of different sphingolipids in MCI and NC groups (**Figure 2**) revealed no significant differences between ceramides (p = 0.47), CerG2GNAc1 (p=0.46), ceramide phosphates (CerP, p=0.67), phosphorylethanolamine ceramides (CerPE, p=0.69), monohexosylceramides (Hex1Cer, p=0.09), dihexosylceramides (Hex2Cer, p=0.62), trihexosyl ceramides (Hex3Cer, p=0.35), sphingomyelins (SM, p=0.92), sphingosine (SP, p=0.8), sphingosine-1-phosphate (SPH, p=0.9) as well as phytosphingomyelins (phSM, p=0.37).

**Figure 3:**
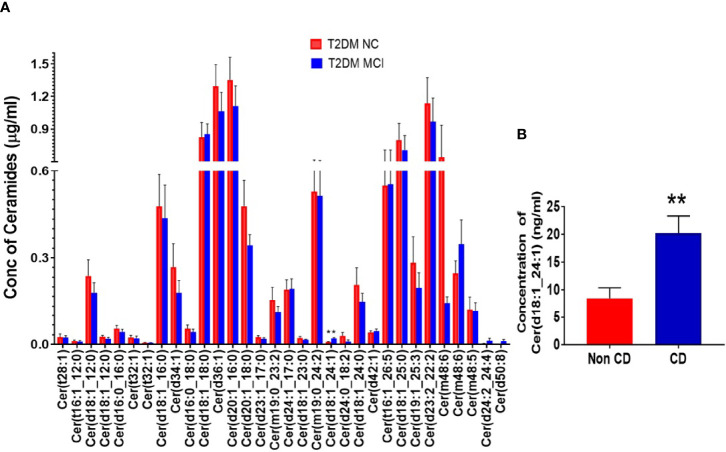
A detailed evaluation of the concentrations of 32 ceramide species individually in the serum type 2 diabetes mellitus patients with mild cognitive impairment (MCI) versus normal cognition (NC). Individual species comparisons were given in **(A)**. This comparison identified significantly higher levels of Cer (d18:1_24:1) [**p < 0.01, **(A, B)**].

**Figure 4:**
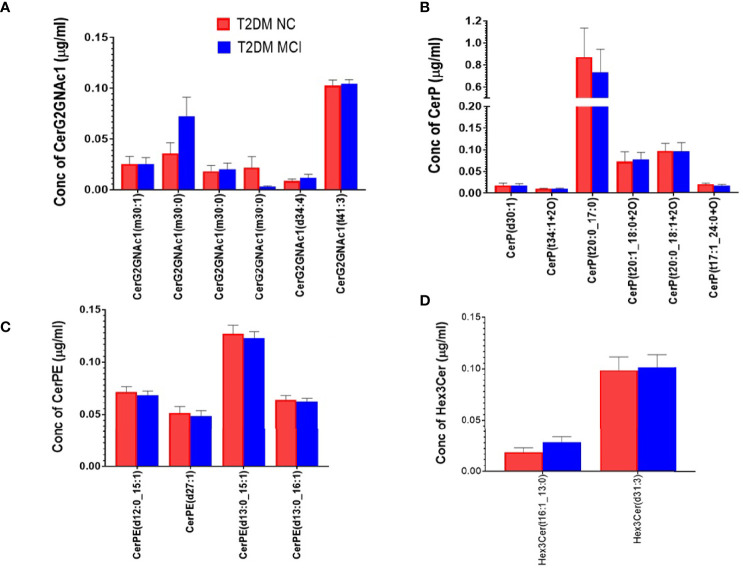
A detailed evaluation of the concentrations of individual species of CerG2GNAc1 species, ceramide phosphates (CerP) and phosphorylethanolamine ceramides (CerPE) and trihexosyl ceramides (Hex 3 Cer) in the serum of MCI and NC group of T2DM patients. Evaluation of 6 species of CerG2GNAc1 **(A)**, 6 species of CerP **(B)**, 4 species of CerPE **(C)**, and 2 species of Hex 3 Cer **(D)** showed no significant difference between the two groups.

A detailed evaluation of the concentrations of 32 ceramide species individually (**Figure 3A**) identified significantly higher levels of Cer (d18:1_24:1) in the serum of MCI compared to the NC group of T2DM patients (p<0.01, **Figures 3A, B**). Analysis of the concentrations of 6 CerG2GNAc1 species (**Figure 4A**), 6 CerP species (**Figure 4B**), as well as 4 CerPE (**Figure 4C**) species showed no significant difference in levels between the two groups.

**Figure 5:**
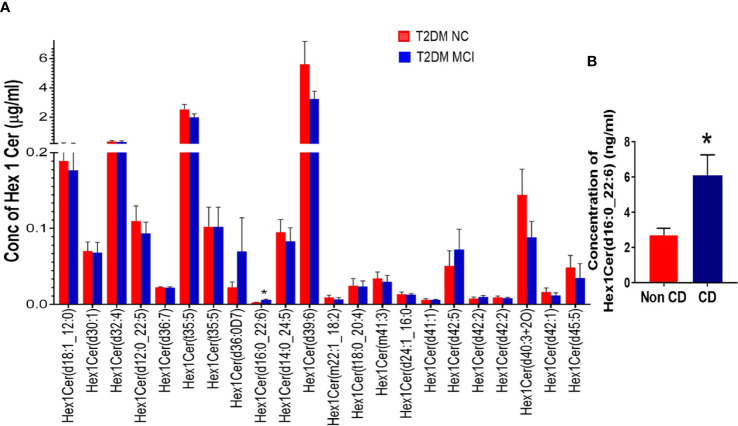
A detailed evaluation of the concentrations of individual species of monohexosylceramides (Hex 1 Cer, 22 species) in patients with MCI compared to the NC group of T2DM patients **(A)**. This analysis identified significantly higher levels of Hex1Cer (d16:0_22:6) in patients with MCI compared to the NC group of T2DM patients [*p < 0.05, **(A, B)**]. Although variations were observed among several other Hex1Cer species, no significant differences were observed between the two groups.

**Figure 6:**
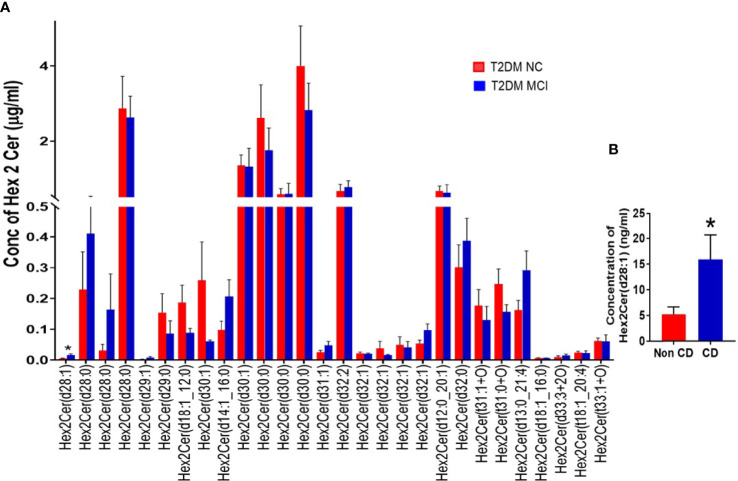
A detailed evaluation of the concentrations of individual species of dihexosylceramides (Hex 2 Cer) in patients with MCI compared to the NC group of T2DM patients **(A)**. Among 28 species of Hex 2 Cer species **(A)**, Hex2Cer (d28:1) levels were significantly higher in the MCI group compared to NC groups [*p < 0.05, **(A, B)**]. Although variations were observed among several other Hex2Cer species, no significant differences were found between the two groups.

Evaluating the concentrations of 22 Hex 1 Cer species individually (**Figure 5A**), identified significantly higher levels of Hex1Cer (d16:0_22:6) in patients with MCI compared to the NC group of T2DM patients NC (p<0.05, **Figures 5A, B**). Among 28 species of Hex 2 Cer species (**Figure 6A**), Hex2Cer (d28:1) levels were significantly higher in the MCI group compared to NC groups (p<0.05, **Figures 6A, B**). Although variations were observed among several other Hex1Cer and Hex2Cer species, no significant differences were demonstrated between MCI and NC groups of T2DM patients. Also, determining the individual concentrations of 2 trihexosyl ceramides (Hex 3 Cer) species demonstrated no significant differences between the two groups (**Figure 4D**).

**Figure 7:**
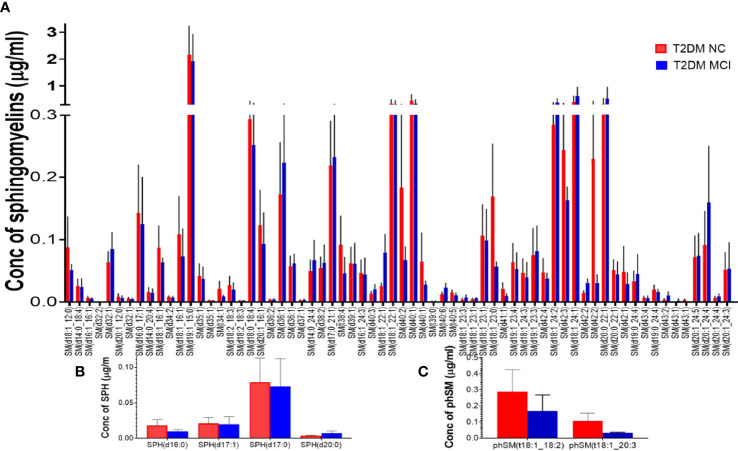
A detailed evaluation of the individual species of sphingomyelins (SM), sphingosine (SPH), and phytosphingomelins (phSM) species in patients with MCI compared to the NC group of T2DM patients. Among 66 SM species evaluated in the targeted lipidomics, none of the species showed significant variations between the two studied groups **(A)**. Similarly, analysis of 4 SPH species **(B)** and 2 phSM species **(C)** demonstrated no significant difference between the two groups.

None of the 66 SM species evaluated in the targeted lipidomics showed significant variations between the two studied groups (**Figure 7A**). Similarly, analysis of 4 SPH species (**Figure 7B**) and 2 phSM species (**Figure 7C**) demonstrated no significant difference between the MCI and NC group of T2DM patients.

## Discussion

Evaluation of cognitive dysfunction in T2DM patients has immense value in preventing the progression to dementia. Through MoCA and DSST, we evaluated MCI in 1278 T2DM patients in a south Indian population and found a prevalence of 35.8%. The study identified a higher incidence of MCI in the female population and an age-dependent increase in the prevalence of MCI in the entire T2DM population. Duration of T2DM over 20 years, higher HbAIc levels, and lower level of education contributed significantly to MCI in the studied T2DM patients. As lipid metabolism is well known to be affected in T2DM, we have done a preliminary analysis of serum lipids through untargeted lipidomics from T2DM patients with MCI and NC. The study mainly identified lower levels of several triglyceride species and phosphatidylcholines containing polyunsaturated fatty acids and higher levels of several sphingolipids in T2DM patients with MCI than the NC group. Given that triglycerides are mainly derived from the diet, and brain-derived sphingolipids could get enriched in the serum due to their higher levels in the brain than the serum, we further quantified sphingolipids from the same serum samples through a targeted lipidomic analysis, which identified 3 species of ceramides, namely Cer (d18:1_24:1), Hex1Cer (d16:0_22:6), Hex2Cer (d28:1) increased in the MCI group compared to NC group of T2DM patients.

Age-related cognitive decline is a well-known phenomenon and 22.2% of the people aged 71 years and above showed cognitive impairment without dementia (22). Gradual decline in short-term memory recall, working memory (23), verbal information recall (24), and spatial memory (25) has been demonstrated with aging (26). Evaluation of cognitive decline through MoCA, a rapid test that assesses cognitive function, has been considered better than the other cognitive tools to detect mild cognitive impairment in T2DM patients (17). The present study evaluated cognitive functions in 1278 T2DM patients through MoCA, which assessed emphasis and attention, executive functions, memory and vocabulary, visuo-constructional capacity, conceptual reasoning, calculations, and orientations. The data were stratified based on age for every 10 years after 40 and until 90, which demonstrated an increasing prevalence of age-related cognitive dysfunction in T2DM patients. The study also evaluated mental speed in these T2DM patients through DSST and demonstrated gradual age-related decline and strong positive correlations with MoCA findings.

The idea of education levels influencing the cognitive decline in aging is a highly debated subject (27, 28). A recent systematic review and meta-analysis downplayed the possibilities of low education influencing age-related cognitive decline (29). Also, structural MRI findings in a longitudinal study revealed that higher education failed to influence brain aging (30). The current study revealed a significantly higher prevalence of MCI in T2DM patients with lower levels of education. The higher prevalence of MCI in this population with lower education could be partly attributed to their inability to personal care in controlling hyperglycemia, as observed from their higher HbA1c levels. Supporting this idea, women lacked good education levels in the study population and displayed a far higher prevalence of MCI than their male counterparts. In a meta-analysis study, the odds of developing vascular dementia in T2DM women were 2.34 times compared to 1.73 times in T2DM men. The odds of having nonvascular dementia in T2DM women were 1.53 times and 1.49 times in T2DM men, implying that diabetic women exhibit 19% higher chances of developing vascular dementia than men (31). The increased propensity to develop MCI in women with T2DM than men with T2DM might be linked to more significant reductions in the hippocampal volume, the region of the brain vital for learning and memory (32). Interestingly, the present study also demonstrated significantly higher levels of total cholesterol in the serum of T2DM females with MCI compared to males. In a previous longitudinal study in aging population, higher basal levels of total cholesterol were associated with increased cognitive decline (33). Midlife high cholesterol levels were also associated with increased risk for late life cognitive decline and dementia (34). Duration of T2DM over 20 years was also an independent factor contributing to cognitive dysfunction in this study. Such finding is expected as longer durations of T2DM is likely associated with prolonged hyperglycemia, impaired glucose tolerance, intermittent drug-mediated hypoglycemia, insulin resistance, chronic inflammation, dysregulation of the hypothalamic-pituitary-adrenal axis, structural, functional, and metabolic brain changes, microvascular complications, which could contribute to cognitive dysfunction (35, 36). Indeed, prolonged hyperglycemia (HbA1c ≥ 7.51) in this study came up as an independent factor contributing to the MCI in T2DM.

Altered carbohydrates (mainly glucose), lipid, and protein metabolism are commonly associated with T2DM. Through untargeted lipidomics, the present study identified significantly lower levels of several triglycerides containing long-chain unsaturated fatty acids and phosphatidylcholines in T2DM patients with MCI than the NC group. Generally, most T2DM patients are advised to control diet, particularly the carbohydrate and lipid-rich diet. Also, several T2DM patients have a presumption to avoid oils and fats in the diet altogether. Such drastic changes in the diet could affect the levels of several TGs and PCs observed in the present study since TG-containing long-chain fatty acids are the essential dietary lipids (37). However, as TGs and PCs could also be synthesized endogenously, further studies are required to dissociate the effects of their deficiency due to a reduced dietary intake vis-à-vis an altered or defective endogenous TG synthesis in T2DM. This is significant as circulating levels of TGs carrying 18-carbon FAs in middle aged adults, demonstrated positive association with cortical thickness, the regions of which are possibly related to cognitive performance (38). Exploring the role of specific long-chain fatty acid-containing TGs is essential as the amount of total triglycerides was unaltered between the MCI and NC groups of T2DM patients in the current study. Although not in T2DM, a recent study reported lower levels of TG-containing long-chain polyunsaturated fatty acids in cases with MCI and AD compared to normal older controls (39). It would be interesting to explore changes in triglycerides containing long-chain fatty acids for their possible role in cognitive function in T2DM.

Emerging evidences suggest the role of ceramides in the pathogenesis of T2DM. Studies in multiple models have demonstrated the crucial role of ceramides in pancreatic β cell function and survival, insulin gene expression, and insulin resistance which acts through multiple mechanisms (40, 41). Ceramides have been implicated in diabetes-induced vascular diseases, diabetic nephropathy, diabetic retinopathy, and diabetic neuropathy (41). The present study identified increased levels of specific ceramides in the serum of T2DM patients with MCI compared to the NC group, implicating their role in cognitive dysfunction. Serum sphingomyelins and ceramides were implicated as early predictors of memory impairment in women (42, 43). Increased levels of serum ceramides were also associated with increased risk for AD in women (44). Further, plasma sphingomyelins and ceramides were considered modifiers of AD risk, and the risk is different for males and females and also based on APOE genotype (45). In a recent study, increase in the ratios of very long-chain (such as C24:0, C22:0) to long-chain (such as C:16) ceramides in the plasma were linked to reduced risk for dementia (46).

The present study provided an early evidence of possible alterations in TG, PC, and sphingolipid levels in MCI in T2DM patients compared to NC groups. Dietary supplementation or restriction of a specific group of lipids could be an opportunity to modify the diet if it helps to improve the cognitive functions in T2DM (47, 48). A recent randomized, placebo-controlled study evaluated the effects of supplementation of medium-chain triglycerides GSK2981710 in healthy older participants for improving cognitive functions in aging (49). In another study, inhibition of specific lipid was demonstrated in an animal model as a therapeutic target to prevent obesity and T2DM (48). In diet supplementation or restriction studies, the focus should be on the critical evaluation of functional measures that are altered due to a dietary change.

In summary, using a cross-sectional study of 1278 T2DM patients, we presented the prevalence of MCI in different age groups of T2DM patients. Also, binomial logistic regression analysis identified the independent factors impairing cognitive function in T2DM patients. Preliminary studies with untargeted lipidomics of the serum from T2DM patients with MCI and NC highlighted reduced levels of several polyunsaturated fatty acid-containing TGs and PCs and increased levels of specific ceramides in the MCI group of T2DM patients. Further analysis with targeted lipidomics identified increased levels of specific ceramides in T2DM patients with MCI. Large-scale lipidomic studies in the future could help understand the cognitive domain in T2DM patients. Furthermore, studies in preclinical models are required to understand the functional significance of the identified lipids in different classes and their association with MCI pathophysiology in T2DM.

## Data Availability Statement

The raw data supporting the conclusions of this article will be made available by the authors, without undue reservation.

## Ethics Statement

The studies involving human participants were reviewed and approved by Kasturba Medical College and Kasturba Hospital Institutional Ethics Committee. The patients/participants provided their written informed consent to participate in this study.

## Author Contributions

DU, MMP, AC, SKP, AKS, KP, RVA and HMH designed the work. AC performed all the prevalence data related work. AC, SH, and CP performed all lipidomic analysis. DU, AC, SH, and SSM analyzed the data. AC and DU wrote the manuscript. MMP, SKP, KP, AKS, and SSM provided their inputs for the manuscript. All authors contributed to the article and approved the submitted version.

## Funding

The study was funded by Research Society for the Study of Diabetes in India (RSSDI/HQ/grants/2019/834). AC and SH are supported by TMA Pai fellowships from Manipal Academy of Higher Education.

## Conflict of Interest

The authors declare that the research was conducted in the absence of any commercial or financial relationships that could be construed as a potential conflict of interest.

## Publisher’s Note

All claims expressed in this article are solely those of the authors and do not necessarily represent those of their affiliated organizations, or those of the publisher, the editors and the reviewers. Any product that may be evaluated in this article, or claim that may be made by its manufacturer, is not guaranteed or endorsed by the publisher.
